# Long Term Exposure to Low Ethylene and Storage Temperatures Delays Calyx Senescence and Maintains ‘Afourer’ Mandarins and Navel Oranges Quality

**DOI:** 10.3390/foods8010019

**Published:** 2019-01-09

**Authors:** Nasiru Alhassan, John B. Golding, Ron B. H. Wills, Michael C. Bowyer, Penta Pristijono

**Affiliations:** 1 School of Environmental and Life Sciences, University of Newcastle, Ourimbah, NSW 2258, Australia; Nasiru.Alhassan@uon.edu.au (N.A.); ron.wills@newcastle.edu.au (R.B.H.W.); michael.bowyer@newcastle.edu.au (M.C.B.); penta.pristijono@newcastle.edu.au (P.P.); 2 NSW Department of Primary Industries, Ourimbah, NSW 2258, Australia

**Keywords:** citrus, ethylene, temperature, calyx senescence, mandarins, oranges

## Abstract

Calyx browning and internal quality loss are major physiological causes for the loss of quality in citrus fruit during storage. While the symptoms of calyx senescence are only superficial, it can affect the appearance and consumer acceptability of citrus fruit. In this study, continuous ethylene exposure at different storage temperatures was investigated to assess their effect on calyx senescence and internal qualities in ‘Afourer’ mandarin and Navel orange fruit during storage. ‘Afourer’ mandarin fruit were stored at ≤0.001 (equivalent to ethylene-free air), 0.01, 0.1 and 1 µL L^−1^ of ethylene at either 5, 10 or 20 °C, whilst in a parallel experiment, Navel oranges were exposed to ≤0.001, 0.1 and 1 µL L^−1^ ethylene at either 1 or 10 °C. Changes in external and internal postharvest quality parameters were assessed for up to 8 weeks for ‘Afourer’ mandarins and 10 weeks for Navel oranges. At all storage temperatures, high levels of ethylene were found to increase the level of calyx senescence, weight loss, loss of fruit firmness and respiration rates. Also, there were significant effects of ethylene and storage temperatures on total soluble solids (TSS) content, titratable acidity (TA), and ethanol accumulation in both citrus species. Continuous exposure to high ethylene also significantly reduced vitamin C and ferric reducing antioxidant power (FRAP) in ‘Afourer’ mandarins after 8 weeks of storage. Overall, ethylene treatments had a significant effect on both the external and internal qualities of the fruit during storage. The relationship between ethylene concentrations and storage temperatures demonstrate that lowering atmospheric ethylene levels at reduced storage temperatures maintain fruit quality during long term storage.

## 1. Introduction

Fruit appearance is an important aspect of consumer purchase and acceptability where the green fresh condition of the fruit calyx (button) is a desirable consumer attribute of citrus appearance [1,2]. Changes in the physical appearance of the calyx can occur as a result of either natural senescence or external stimuli such as heat stress, causing colour change (from green to yellow and brown), with abscission of the calyx. Retention of the calyx is important not only because it enhances cosmetic appeal, but it also protects against fungal infection in the abscission zone of the fruit peduncle and extends fruit storage life [2]. Both mandarins and to a lesser extent oranges are susceptible to calyx senescence during storage.

Citrus fruit can be stored for up to 8 weeks under optimal conditions, but storage life depends on pre-harvest conditions, cultivar and storage environment, where lower storage temperatures reduce fruit senescence [3]. However citrus fruit can be sensitive to chilling injury (CI) when exposed to chilling temperatures (<3 °C to 5 °C) depending on citrus type and cultivar.

Citrus fruit are considered non-climacteric, but are responsive to postharvest application of ethylene [3], which has been shown to induce a desirable colour change in citrus fruit, and is widely used to commercially degreen early season fruit [4,5]. A short-term ethylene treatment (4 days with 2 μL L^−1^ ethylene) has also been shown to protect fruit from stress related postharvest disorders such as CI and non-chilling peel pitting [6]. Storage of citrus fruit in an ethylene environment at low temperature (0 °C) has been shown to reduce the development of CI symptoms [7]. However, ethylene has also been shown to produce undesirable effects related to accelerated fruit senescence including abscission of the calyx, calyx browning and calyx drying [8]. Brown and Burns [9] found that fruit exposure to ethylene (55 μL L^−1^) during degreening induced calyx senescence in ‘Valencia’ oranges and increased calyx abscission. However, studies by Li et al. [10] showed that when ethylene levels were decreased from 1 to <0.001 μL L^−1^, the incidence of calyx senescence in ‘Afourer’ mandarins diminished. The development of undesirable characteristics in citrus as a consequence of ethylene exposure varies with citrus type and cultivar [11]. Lafuente et al. [6] found that while an ethylene concentration of 2 µL L^−1^ at 2 °C storage reduced calyx abscission in ‘Lane Late’ oranges, the same treatment conditions for ‘Navelate’ oranges resulted in high calyx abscission.

Many studies on the effects of ethylene on citrus fruit quality have been conducted examining the effects of commercial degreening treatments, where high levels of ethylene (up to 4 μL L^−1^) are applied for a short period of time (a few days) [12,13,14]. These short-term degreening treatments are not comparable to longer treatment and storage times, where fruit can be exposed to low levels (<1 μL L^−1^) of ethylene during storage [10].

Ethylene exposure at low concentrations during storage has been linked to compositional changes of citrus [15,16]. Li et al. [10] observed lower ethanol levels in ‘Afourer’ mandarins during prolonged storage (10 weeks) in a low ethylene atmosphere while Mahler et al. [17] found no effect on total soluble solids (TSS) and titratable acidity (TA) levels in ‘Navelina’ orange stored under 0.5 µL L^−1^ ethylene at 5 or 20 °C. Accumulation of volatiles such as ethanol is often associated with off-flavour sensory qualities, particularly in citrus juice products [18,19] and must therefore be avoided during long-term storage.

There are few reports on assessing the effect of long-term ethylene exposure on calyx condition and internal quality attributes of mandarins and oranges held in long-term storage (cold or ambient temperature) [6,10]. The few cited studies conducted have not undertaken an extensive evaluation of the impact of ethylene concentrations and storage temperature on fruit quality parameters, including vitamin C content and antioxidant activity. The objective of this study was to assess the impact of four ethylene concentrations and different storage temperatures on calyx senescence and internal quality parameters of ‘Afourer’ mandarins (also known as ‘Nadorcott’ or ‘W. Murcott’) and Navel oranges during long-term storage.

## 2. Materials and Methods

### 2.1. Plant Materials and Experimental Procedure

Mature mandarin fruit (*Citrus reticulata* Blanca cv. ‘Afourer’) were obtained from a commercial packinghouse in Gayndah, Queensland, Australia (25°38′00″ S, 151°35′47″ F). Fruit used for this experiment had their calyx intact, were waxed with commercial carnauba wax and were not treated with the growth regulator 2,4-dichlorophenoxyacetic acid (2,4-D). Fruit for the experiment were uniform in size and free from visible defects. Fruit were randomized and placed into 48 replicate net bags (*n* = 20 fruit per bag). The bags were then evenly distributed into 6.3 L plastic containers (*n* = 16) fitted with inlet and outlet airflow ports and stored at either 5, 10 or 20 °C. Individual containers within each temperature environment were then connected to one of four ethylene containing humidified air streams (≤0.001, 0.01, 0.1 and 1 μL L^−1^) operating at a flow rate of 50 mL min^−1^. The ≤0.001 µL L^−1^ stream was considered to be ethylene-free (air) as the ethylene concentration is below the limit of detection by gas chromatography. The experiment was monitored daily. Mandarin fruit stored at 20 °C were assessed as weekly removals for four weeks, while fruit stored at 10 and 5 °C were assessed every two weeks for eight weeks.

In a parallel study, Navel oranges (*Citrus sinensis* L. Osbeck) were harvested at commercial maturity from a New South Wales Department of Primary Industries (NSW DPI) research farm at Somersby on the NSW Central Coast, Australia (33°36′50″ S, 151°28′50″ F). Fruit were transported to NSW DPI postharvest laboratories (Ourimbah, NSW, Australia) (10 min), where the fruit was sanitised with commercial sodium hydrochlorite solution (50 ppm) before being washed and air dried. The fruit was then sized and sorted as previously described, randomized and placed in net bags as individual treatment units (*n* = 20 fruit). The treatment units were then placed in 60 L steel drums and stored in two different temperatures (1 and 10 °C). The individual drums within each temperature were connected to one of the four humidified air streams containing ethylene (≤0.001, 0.1 and 1 µL L^−1^) operating at a flow rate of 400 mL min^−1^. The drums were inspected daily. There were four replicates (in different drums, i.e., independent replicates) for each treatment with assessments after 1, 5 and 10 weeks at both storage temperatures and again with an additional shelf-life assessment following 5 days storage at 20 °C.

### 2.2. Physio-Chemical Assessments

#### 2.2.1. Calyx Senescence and Chilling Injury Assessment

Calyx senescence was visually evaluated with changes in colour of the calyxes from green to brown and scored according to Li et al. [10] using a 5-point scale where 1 = green, 2 = slightly yellow, 3 = moderately yellow, 4 = totally yellow and 5 = brown and the mean score of all fruit in the sample then calculated. Calyx abscission was assessed by the presence or absence of the calyx of the fruit. Calyxes which had abscised during storage were counted on each assessment day and the results expressed as percentage calyx abscission. Chilling injury (CI) was assessed using a 4 point scale previously described by Lafuente and Sala [20] where 0 = normal (no pitting symptoms), 1 = slight pitting (a few scattered pits), 2 = moderate pitting (up to 30% surface covering), 3 = severe pitting (>30% surface covering), with results expressed as a percentages of the total number of fruit evaluated in the experiment.

#### 2.2.2. Weight Loss

Weight loss of the fruit was assessed using an electronic balance (Model Kern & Sohn GmbH, D-72336, Balingen, Germany), where fruit weight of each treatment unit was recorded each assessment day. Weight change was expressed as a percentage value determined by deducting the initial weights (W_1_) from the final weights (W_2_) divided by the initial weights and multiplied by hundred percent (%).

#### 2.2.3. Measurement of Fruit Firmness

A texture analyser (Lloyd Instrument LTD, Fareham, UK) was used to determine firmness of ten fruit per replicate after the fruit had conditioned to 20 °C. The maximum force (N) was measured by compressing the fruit in the equatorial zone between two flat surfaces closing together at the rate of 1 mm min^−1^ to a depth of 2 mm. The average of two reading points from each side of the fruit was recorded [21].

#### 2.2.4. Respiration Rate

Respiration rate (mL CO_2_ kg^−1^ h^−1^) was measured on five fruit from each replicate. Fruit were sealed into an airtight 2 L glass jars fitted with a septum for 3 h to accumulate respiratory gases. Carbon dioxide (CO_2_) concentration in the jar was determined by withdrawing a 1 mL gas sample from the headspace and injecting into a gas chromatograph (Gow-Mac, Bridgewater, NJ, USA) fitted with two stainless steel columns (60 cm × 1 mm i.d.) connected in series. Operating temperatures for the detector, injector, and column were 110 °C, 50 °C and 110 °C respectively. The carrier gas used was high purity argon (BOC Gases, Sydney, NSW, Australia) at a flow rate of 25 mL min^−1^. Respiration rate was calculated according to the method of Huque et al. [22].

#### 2.2.5. Internal Fruit Quality Assessments

Two fruit from each replicate were manually juiced and sieved through two layers of cheesecloth. TSS content of the juice was determined using a digital refractometer (Atago Co., Ltd., Tokyo Atago, Japan) and expressed as °Brix. TA was determined by titrating 5 mL of the juice with 0.1 M NaOH to pH 8.2 with an automatic titrator (Mettler Toledo, Greifensee, Switzerland) and data were expressed as percentage citric acid. For ethanol accumulation, 10 mL of juice was transferred into a 20 mL glass vial with crimp-top caps sealed with silicone septa. The sealed sample was then incubated in a water bath of 30 °C for 10 min before analysis. Ethanol accumulation was determined by headspace analysis using a gas chromatograph (Model 580, Gow-Mac, Bethlehem, PA, USA) with a flame ionization detector and a column (Carbwax, Gow-Mac, Bethleham, PA, USA). The injector was set at 190 °C, the column at 68 °C, the detector (FID) at 190 °C with gas flow rates of 30, 30 and 300 mL min ^−1^ for nitrogen, hydrogen and air respectively. After incubating the samples, 1 mL of the headspace gas sample was drawn from the vials and injected into the GC. Ethanol accumulation was calculated and expressed as g L^−1^.

#### 2.2.6. Vitamin C Content

Ascorbic acid was determined by the indophenol titration method adapted from the method of Nielsen et al. [23]. Briefly, 10 mL of fruit juice was extracted from fruit and filtered through two layers of cheesecloth. Five (5) mL of metaphosphoric acid-acetic acid solution was then added to the filtered extract and the samples then titrated with the 2,6-dichloroindophenol dye solution until samples turned light rose-pink colour. The quantity of dye used in the titration was then used to calculate the vitamin C content present.

#### 2.2.7. Antioxidant Activity

The antioxidant activity of the ‘Afourer’ mandarin fruit were determined using ferric reducing antioxidant power (FRAP) assay as described by Thaipong et al. [24], with some modification. A working FRAP solution was prepared by mixing 300 mM acetate buffer, 10 mM 2,4,6-Tris(2-pyridyl)-*s*-triazine (TPTZ) reagent in 40 mM HCl and 20 mM ferric chloride in the ratio of 10:1:1 and warmed to 37 °C in a water bath. 2.85 mL of the FRAP working solution was then added to a 150 mL aliquot of mandarin juice and the resulting solution allowed to react in the dark for 30 min at 20 °C. The solution absorbance (λ = 593 nm) was then recorded using a UV-Vis spectrophotometer (Varian Australia Pty. Ltd., Melbourne, VIC, Australia) and the result referenced against a standard curve and expressed as µmol of Trolox equivalents (TE) per 100 mL fresh mandarin juice.

#### 2.2.8. Statistical Analysis

Data from each of the experiments were subjected to two-way analysis of variance (ANOVA) using SPSS Microsoft version 24.0 software package (SPSS, Chicago, IL, USA). Tests for significance difference of treatment means were at *p* ≤ 0.05. The method used to discriminate among the means (Multiple Range Test) was Fisher’s Least Significance Difference (LSD) procedure at 95% confidence level.

## 3. Results and Discussion

### 3.1. Changes in External Quality

#### 3.1.1. Calyx Browning

External fruit appearance is an important characteristic that influences consumer purchasing decisions. The results of these experiments showed a significant effect of both ethylene concentration and storage temperature (*p* < 0.001) on calyx browning during storage. However no significant interaction was observed between ethylene concentration and storage temperature. The data presented (Table 1 and Table 2) show the levels of calyx browning increased with both increasing ethylene concentration and storage temperature. Storage at low ethylene concentrations (≤0.1 µL L^−1^) resulted in reduced calyx browning and maintained visible quality. Fruit at stored 5 and 1 °C under low atmospheric ethylene concentration (≤0.1 µL L^−1^) showed reduced calyx browning compared with the fruit stored at 20 and 10 °C. These results with ‘Afourer’ mandarins and Navel oranges are in agreement with the findings of Li et al. [10] who also reported that continuous low ethylene levels reduced calyx senescence in ‘Afourer’ mandarins.

#### 3.1.2. Calyx Abscission

There was a significant interaction between ethylene and storage temperature on the incidence of calyx abscission during storage. Both Navel oranges and ‘Afourer’ mandarins had higher levels of calyx abscission at increased ethylene levels and storage temperatures. The data presented in Table 1 and Table 2 show that calyx abscission was greater in fruit stored at 20 and 10 °C with large decreases observed at 5 and 1 °C for ‘Afourer’ mandarins and Navel oranges. The incidence of calyx abscission was highest in the fruit exposed to ethylene levels of 1 µL L^−1^. Higher calyx abscission levels were observed in the ‘Afourer’ mandarins compared to the Navel oranges, which was due to physiological differences between the citrus species. However these observations were dependent on the interaction with ethylene concentration and storage temperature. Air treatments (i.e., ethylene at <0.001 µL L^−1^) at different temperatures resulted in significantly lower calyx abscission compared to the other ethylene concentrations. These results support previously published findings showing that lower ethylene levels (‘air’ or <0.1 µL L^−1^) and low storages temperatures (<10 °C) reduce calyx drop in Navelate orange fruit and ‘Afourer’ mandarins [6,10]. The results of this study also supports the findings of Brown and Burns [9] who reported that a short term exposure to high levels of ethylene during degreening induced calyx senescence and increased calyx abscission in Valencia oranges.

#### 3.1.3. Fruit Firmness

There was a significant effect of both ethylene treatment and storage temperature (*p* < 0.001) and their interaction on the firmness of ‘Afourer’ mandarins and Navel oranges during storage. The results presented (Table 1 and Table 3) show fruit treated with 1 µL L^−1^ ethylene had lower fruit firmness (i.e., were softer), while fruit exposed to an ethylene concentration of <0.1 µL L^−1^ at all storage temperatures recorded the highest firmness level. There was a significant interaction with ethylene concentration and storage temperature, where fruit firmness levels were lower in fruit stored at higher temperatures for both citrus types (Table 1 and Table 3). This observation confirms the report by Lafuente et al. [6] who showed that fruit firmness in Lane’s Late oranges stored for 20 days at 12 °C decreased relative to Navelate oranges stored at 2 °C. The decrease in fruit firmness in the presence of ethylene may be due to increased metabolic activity (respiration) (Section 3.2.1). This is contrary to work by Sdiri et al. [11] who found firmness of ‘Clemenules’ mandarins was slightly increased by ethylene degreening. However these degreening treatments [11] use only a short-term ethylene treatment (few days) before returning the fruit to air for the rest of the storage period and assessment.

#### 3.1.4. Chilling Injury

There was significant effect of both ethylene and storage temperature (*p* < 0.001) and their interaction on chilling injury (CI) of Navel oranges. No CI symptoms were observed in ‘Afourer’ mandarins stored at 5 °C. Storage of Navel oranges at 1 °C produced CI symptoms in Navel oranges after 5 weeks of storage followed by 5 days at 20 °C (Table 2). These symptoms only generally appeared after the addition of the 5 days shelf life. No incidence of CI was found on the Navel oranges held at 10 °C for all ethylene concentrations during the experiment, as this storage temperature is not a chilling temperature for citrus [25]. At the end of the storage and shelf life simulation, Navel oranges exposed to all ethylene levels at 1 °C were affected by CI, but the symptoms were most severe in fruit stored at low ethylene levels (≤0.1 µL L^−1^). It is known that ethylene treatment at low storage temperature plays a synergistic role in the maintenance of cell integrity, particularly in cell membranes under stress [26,27], so at the higher ethylene concentrations and low storage temperatures the CI symptom was lower (Table 2). This observation is in agreement with Lafuente et al. [6] who showed that ethylene treatment combined with low storage temperature reduced CI in Navelate oranges. The results in this work also supports the findings that citrus fruit stored at low temperature (0 °C) and at reduced levels of exogenous ethylene were found to develop earlier and more severe CI in different citrus fruit [7].

#### 3.1.5. Fruit Weight Loss

There was significant (*p* < 0.001) interaction of storage temperature and ethylene on the weight loss of both ‘Afourer’ mandarins of Navel oranges during storage (Table 3 and Table 4). The highest percentage of water loss was observed for fruit stored in highest ethylene atmosphere (1 µL L^−1^) with weight loss also increasing with increasing storage temperature. Conversely, lower levels of weight loss were observed in fruit stored in low ethylene concentrations at low temperatures, which can be attributed to reduced transpiration and metabolic activity.

### 3.2. Changes in Respiration Rate and Ethanol Accumulation in Fruit

#### 3.2.1. Respiration Rate

There was significant effect of both ethylene concentrations and temperature (*p* < 0.001) on the respiration rate of ‘Afourer’ mandarins and Navel oranges during storage but there was no significant interaction between ethylene concentration and storage temperature. The data presented (Table 3 and Table 4) shows that fruit exposed to lower ethylene concentrations (<1 µL L^−1^) at all the storage temperatures reduced respiration rate. In both citrus species, exposure to ethylene-free air (<0.001 µL L^−1^) resulted in the lowest respiration rate within each storage temperature during storage. However, a differential response to ethylene exposure and temperature was observed, with Navel oranges exhibiting a significantly lower respiration rate compared with ‘Afourer’ mandarins exposed to similar ethylene concentrations and storage temperature (10 °C) (Table 3 and Table 4). Although citrus is generally classified as non-climacteric fruit and have characteristically low respiration rates, conditions such as the presence of low levels of ethylene have significant effects on the fruit respiration rate, which can in turn have adverse effects on other quality parameters.

#### 3.2.2. Ethanol Accumulation

The occurrence of off-flavors in stored citrus fruit has been linked to the higher accumulation of volatiles such as ethanol [18]. In these experiments, there was significant effect of both ethylene concentrations and storage temperature (*p* < 0.001) on the ethanol accumulation in ‘Afourer’ mandarins and Navel oranges. The data presented (Table 4 and Table 5) show that ethanol accumulation increased with increasing ethylene concentrations in both fruit species. Mandarin fruit stored at 20 and 10 °C had higher ethanol accumulation compared with fruit at 5 °C (Table 4). Overall, fruit stored at 5 and 1 °C for both citrus types recorded low ethanol levels during the experiments. The concentration of ethanol in the ‘Afourer’ mandarins was found to be higher than in Navel oranges at the end of the storage period (Table 4 and Table 5). The result is consistent with previous findings which show that mandarin fruit are more susceptible to the development of strong off-flavor characteristics in comparison with to other citrus varieties [19]. Ethanol accumulation exhibited a concentration dependent relationship with the level of atmospheric ethylene at all storage temperatures. This finding is consistent with the work of Mahler et al. [17] who found that Navel oranges stored at 5 °C and exposed to exogenous ethylene recorded significantly higher levels of ethanol compared with the untreated controls.

### 3.3. Changes in Total Soluble Solids, Titratable Acidity and Vitamin C Content

#### 3.3.1. Total Soluble Solids

There was a significant effect of both ethylene and storage temperature (*p* < 0.05) on total soluble solids (TSS) in both mandarins and oranges (Table 5 and Table 6). While differences in TSS levels were found to be statistically different, the magnitude of these differences are not commercially significant (i.e., <1% Brix). The small differences noted similarly compared with Mahler et al. [17] who found no effect on TSS in ‘Navelina’ oranges exposed to ethylene at two storage temperatures (5 or 20 °C) and with Li et al. [10] who reported no significant effect of ethylene levels and storage temperatures on TSS of ‘Afourer’ mandarins during 10 weeks storage.

#### 3.3.2. Titratable Acidity

In ‘Afourer’ mandarins and Navel oranges, both ethylene concentrations and storage temperatures had a significant effect (*p* < 0.001) on titratable acidity (TA), with no significant interaction between these factors. The TA of both ‘Afourer’ mandarins and Navel oranges decreased during storage, with ‘Afourer’ mandarins exhibiting lower TA levels than in Navel oranges. The data presented (Table 5 and Table 6) shows that TA level decreased with increasing in ethylene concentrations in both citrus types. However, these results contrast to other reports which report no effect of continuous ethylene treatment on TA levels on Navelina orange stored at either 5 or 20 °C [17].

#### 3.3.3. Vitamin C Content

The concentrations of vitamin C (L-ascorbic acid) in ‘Afourer’ mandarins showed a significant interaction between storage temperature and ethylene treatment (*p* < 0.05). The average vitamin C level at the beginning of the experiment was 7.1 mg 100 mL^−1^ (Table 6). As expected, vitamin C levels fell with storage time across all storage temperatures, with the effect tempering as ethylene concentration and temperature was lowered. The results presented in Table 6 show that all ethylene treatments at 5 and 10 °C retained higher vitamin C content at the end of storage compared with fruit stored at 20 °C, particularly at lower ethylene levels. The decrease in vitamin C levels with increases in storage temperature is widely observed in citrus fruit [12,13]. Vitamin C content and overall antioxidant capacity of ‘Clemenpons’ Clementine mandarins has been shown not to be affected by short-term ethylene degreening treatment in storage [11,14]. However in this long-term storage experiment, the continuous presence of ethylene in the storage atmosphere had an effect the vitamin C degradation.

### 3.4. Antioxidant (FRAP) Activity in Mandarins

Ferric reducing antioxidant power (FRAP) assay measures the antioxidant potential through the reduction of ferric ion (Fe^3+^) and ferrous ion (Fe^2+^) by antioxidants present in the sample [28,29]. In this experiment, there was significant interaction between ethylene concentrations and storage temperature (*p* < 0.001) in relation to antioxidant activity in ‘Afourer’ mandarins during storage with results showing a decrease in FRAP levels across the storage period. There was also a significant decrease in activity observed in fruit exposed to 1 µL L^−1^ ethylene at all storage temperatures (Table 7). This contrasts with the findings of Mayuoni et al. [14] who reported no significant changes in the antioxidant capacity of ‘Clemenpons’ Clementine mandarin during a short-term ethylene degreening at 20 °C storage temperature. In this experiment, the FRAP antioxidant activity was adversely affected at higher ethylene concentrations and storage temperatures compared with those fruit held at low storage temperature of 5 °C.

## 4. Conclusions

Effects on calyx senescence and postharvest quality of ‘Afourer’ mandarins and Navel oranges were investigated as a function of ethylene concentration and storage temperatures. The results showed that continuous exposure to lower ethylene concentrations (≤0.01 µL L^−1^) significantly reduced calyx senescence changes (i.e., browning and abscission). Lower ethylene concentrations at all storage temperatures were also found to be beneficial to most postharvest quality factors. However the primary factor limiting the storage life of ‘Afourer’ mandarins and Navel oranges was storage temperature. For both citrus species, storage temperatures of 5 and 1 °C exhibited the slowest calyx browning and abscission. Storage at 5 °C under low ethylene concentration (≤0.1 µL L^−1^) was found to be more beneficial to calyx senescence than other storage temperatures. Ethanol accumulation in fruit during storage is known to contribute to the development of off-flavours. In this experiment, ethanol production was the higher in ‘Afourer’ mandarin than Navel oranges and storage with high atmospheric ethylene concentrations contributed to higher ethanol levels in the juice. In ‘Afourer’ mandarins, continuous exposure to low levels of ethylene also had a significant effect on both vitamin C concentration and ferric reducing antioxidant power (FRAP) after 8 weeks of storage. Chilling injury was associated with low ethylene concentrations, which only occurred on Navel oranges held at 1 °C. Further studies assessing the effects of low ethylene concentrations and storage temperature on calyx senescence and CI need to be conducted on other citrus species and cultivars to obtain a more comprehensive understanding of individual behavior.

## Figures and Tables

**Table 1:** Calyx browning, calyx abscission and firmness level in ‘Afourer’ mandarins exposed to different ethylene concentrations and stored at 20, 10 and 5 °C.

Quality Parameter/Ethylene Level (µL L^−1^)	Storage Temperature/Storage Time (Weeks)												Mean
	20 °C				10 °C				5 °C				
	1	2	3	4 Weeks	2	4	6	8 Weeks	2	4	6	8 Weeks	
Calyx browning score													
Time-0	2.0												
<0.001	2.6	3.1	3.4	4.0	2.5	2.9	3.1	3.5	2.3	2.6	2.9	3.4	3.0 ^a^
0.01	2.9	3.3	3.7	4.3	2.6	3.0	3.3	3.9	2.5	2.8	3.0	3.6	3.4 ^b^
0.1	3.0	3.5	3.9	4.4	2.7	3.2	3.6	4.2	2.6	2.9	3.2	3.8	3.4 ^b^
1	3.6	4.3	4.7	4.8	3.2	3.6	4.3	4.6	2.9	3.4	3.7	4.4	4.0 ^c^
Mean	3.7 ^a^				3.4 ^b^				3.1 ^c^				
LSD	0.1												0.1
Calyx abscission (%)													
Time-0	0												
<0.001	20	37	42	52	17	25	32	37	12	20	22	25	28 ^a^
0.01	32	42	45	57	22	32	35	42	20	25	30	35	35 ^b^
0.1	35	45	47	62	25	35	37	47	22	30	35	40	38 ^c^
1	42	50	60	72	37	45	47	60	30	35	37	45	47 ^d^
Mean	46 ^a^				35 ^b^				29 ^c^				
LSD	2												2.3
Firmness level (N)													
Time-0	14.7												
<0.001	14.2	13.1	11.9	11.6	13.3	12.7	12.4	12.3	12.9	12.5	11.9	11.3	12.5 ^a^
0.01	13.9	12.4	11.2	11.0	14.2	13.2	12.1	12.6	13.0	12.6	12.3	12.2	12.6 ^ab^
0.1	14.0	12.7	11.5	11.0	14.3	13.5	12.9	12.8	13.3	12.7	12.7	12.5	12.8 ^b^
1	13.7	12.2	10.5	9.9	13.1	12.3	11.9	10.9	12.6	11.5	11.1	10.5	11.7 ^c^
Mean	11.5 ^a^				12.8 ^b^				12.2 ^c^				
LSD	0.2												0.3

Values are the means of 4 replicates. Means with the same superscript letter across each column and row are not significantly different at *p* ≤ 0.05. LSD: Least Significance Difference.

**Table 2:** Calyx browning, calyx abscission and chilling injury of Navel oranges stored at 10 and 1 °C.

Quality Parameter/Ethylene Levels (µL L^−1^)	Storage Temperature/Storage Time (Weeks)						Mean
	10 °C			1 °C			
	1	5	10 Weeks	1	5	10 Weeks	
Calyx browning score							
Time-0	1.0						
<0.001	1.6	2.0	2.7	1.5	1.8	2.3	2.00 ^a^
0.1	1.7	2.2	3.0	1.6	1.9	2.5	2.20 ^b^
1	1.9	2.3	3.7	1.9	2.0	2.7	2.40 ^c^
Mean	2.30 ^a^			2.00 ^b^			
LSD	0.03						0.04
Calyx abscission (%)							
Time-0	0						
<0.001	17	32	46	6	15	24	23 ^a^
0.1	20	40	55	9	20	32	29 ^b^
1	26	49	61	14	27	41	36 ^c^
Mean	38 ^a^			21 ^b^			
LSD	4						3
Chilling injury score							
Time-0	0						
<0.001	0	0	0	0.89	1.76	3	0.94 ^a^
0.1	0	0	0	0.58	1.13	2	0.62 ^b^
1	0	0	0	0.34	0.7	1.21	0.38 ^c^
Mean	0.00 ^a^			1.29 ^b^			
LSD	0.04						0.1

Values are the means of 4 replicates. Means with the same superscript letter across each column and row are not significantly different at *p* ≤ 0.05.

**Table 3:** Weight loss, firmness and respiration rate of Navel oranges stored at 1 and 10 °C.

Quality Parameter/Ethylene Levels (µL L^−1^)	Storage Temperature/Storage Time (Weeks)						Mean
	10 °C			1 °C			
	1	5	10 Weeks	1	5	10 Weeks	
Weight loss (%)							
Time-0	0						
<0.001	2.8	3.8	4.3	3.2	3.7	4.7	3.8 ^a^
0.1	3.1	4.3	5.0	2.6	3.2	4.1	3.7 ^a^
1	3.3	4.5	5.5	2.9	3.4	4.3	4.0 ^a^
Mean	4.1 ^a^			3.6 ^b^			
LSD	0.4						0.4
Firmness level (N)							
Time-0	51.3						
<0.001	35.6	28.8	27.6	38.9	33.1	29.0	32.2 ^a^
0.1	34.3	27.7	26.0	41.0	36.2	33.2	33.1 ^b^
1	33.1	25.6	23.6	38.9	34.6	31.3	31.2 ^c^
Mean	29.1 ^a^			35.1 ^b^			
LSD	0.6						0.7
Respiration rate (mL CO_2_ kg^−1^ h^−1^)							
Time-0	7.0						
<0.001	8.5	12.9	16.0	8.1	12.7	13.8	12.0 ^a^
0.1	9.3	14.5	16.4	9.0	14.8	14.9	13.2 ^b^
1	9.6	14.7	16.8	10.3	15.2	15.3	13.7 ^c^
Mean	13.2 ^a^			12.7 ^a^			
LSD	1.0						1.2

Values are the means of 4 replicates. Means with the same superscript letter across each column and row are not significantly different at *p* ≤ 0.05.

**Table 4:** Weight loss, respiration rate and ethanol accumulation in ‘Afourer’ mandarins as exposed to different ethylene concentrations and stored at 20, 10 and 5 °C.

Quality Parameter/Ethylene Level (µL L^−1^)	Storage Temperature/Storage Time (Weeks)												Mean
	20 °C				10 °C				5 °C				
	1	2	3	4 Weeks	2	4	6	8 Weeks	2	4	6	8 Weeks	
Weight loss (%)													
Time-0	0												
<0.001	0.52	0.77	1.26	1.68	0.74	1.12	1.60	2.26	0.47	0.54	0.53	0.78	1.02 ^a^
0.01	0.55	0.79	1.63	1.84	0.68	1.10	1.58	2.25	0.46	0.44	0.45	0.77	1.05 ^a^
0.1	0.57	0.87	2.30	1.87	0.66	0.95	1.35	2.08	0.42	0.39	0.29	0.74	1.04 ^a^
1	0.66	1.53	4.38	2.26	1.00	1.17	3.56	2.46	0.57	0.46	0.48	0.78	1.61 ^b^
Mean	1.47 ^a^				1.54 ^b^				0.54 ^c^				
LSD	0.9												0.3
Respiration rate (mL CO_2_ kg^−1^ h^−1^)													
Time-0	10.9												
<0.001	23.6	28.5	28.6	28.9	24.0	26.0	25.0	26.5	12.6	17.3	18.1	23.7	23.6 ^a^
0.01	24.6	29.1	32.5	32.4	24.7	25.3	26.6	30.9	13.9	19.4	19.9	29.7	25.8 ^b^
0.1	26.8	32.7	33.2	33.8	26.8	27.7	28.4	31.8	15.2	21.1	21.7	15.4	26.2 ^b^
1	27.6	33.4	33.8	34.8	28.0	30.7	32.2	32.9	17.6	23.6	24.4	31.7	29.2 ^c^
Mean	30.3 ^a^				28.0 ^b^				20.3 ^c^				
LSD	1.3												1.6
Ethanol production (g L^−1^)													
Time-0	0.2												
<0.001	0.6	0.9	1.2	2.0	0.8	0.8	1.3	2.0	0.3	0.4	0.5	0.6	0.95 ^a^
0.01	0.6	1.1	1.3	2.3	0.9	0.8	1.5	2.3	0.4	0.5	1.1	1.5	1.19 ^a^
0.1	0.6	1.5	1.5	2.4	1.5	1.5	1.5	2.3	0.3	0.6	0.4	0.5	1.22 ^a^
1	0.5	1.3	1.5	2.2	0.5	0.5	1.6	2	0.5	1	1.5	1.9	1.25 ^b^
Mean	1.3 ^a^				1.4 ^b^				0.8 ^b^				
LSD	0.2												0.3

Values are the means of 4 replicates. Means with the same superscript letter across each column and row are not significantly different at *p* ≤ 0.05.

**Table 5:** TSS, TA and ethanol accumulation of Navel oranges stored at 1 and 10 °C.

Quality Parameter/Ethylene Levels (µL L^−1^)	Storage Temperature/Storage Time (Weeks)						Mean
	10 °C			1 °C			
	1	5	10 Weeks	1	5	10 Weeks	
TSS (%)							
Time-0	11.0						
<0.001	12.0	12.7	13.5	11.4	11.7	12.2	12.3 ^a^
0.1	11.7	11.7	12.5	11.3	11.4	11.8	11.7 ^b^
1	11.2	11.3	11.6	11.1	11.2	11.5	11.3 ^c^
Mean	12.0 ^a^			11.5 ^b^			
LSD	0.2						0.3
TA (% citric acid)							
Time-0	1.26						
<0.001	1.26	1.24	1.21	1.25	1.23	1.17	1.23 ^a^
0.1	1.25	1.22	1.15	1.23	1.21	1.14	1.20 ^ab^
1	1.23	1.20	1.08	1.20	1.16	1.04	1.15 ^b^
Mean	1.20 ^a^			1.18 ^b^			
LSD	0.07						0.08
Ethanol accumulation (g L^−1^)							
Time-0	0.80						
<0.001	0.83	0.99	1.01	0.85	1.04	1.17	0.98 ^a^
0.1	0.90	1.17	1.29	0.80	0.97	1.10	1.04 ^a^
1	1.01	1.36	1.60	0.95	1.10	1.21	1.21 ^b^
Mean	1.13 ^a^			1.02 ^a^			
LSD	0.13						0.16

Values are the means of 4 replicates. Means with the same superscript letter across each column and row are not significantly different at *p* ≤ 0.05.

**Table 6:** TSS, titratable acidity (TA) and vitamin C content of ‘Afourer’ mandarins during storage at 20, 10 and 5 °C.

Quality Parameter/Ethylene Level (µL L^−1^)	Storage Temperature/Storage Time (Weeks)												Mean
	20 °C				10 °C				5 °C				
	1	2	3	4 Weeks	2	4	6	8 Weeks	2	4	6	8 Weeks	
TSS (%)													
Time-0	10.2												
<0.001	9.8	10.1	10.2	10.5	9.5	10.1	9.8	9.6	8.2	9.4	9.5	9.6	9.7 ^a^
0.01	9.9	9.0	9.8	10.1	9.7	9.9	9.6	9.3	8.0	9.6	9.5	9.8	9.5 ^b^
0.1	10.0	8.8	9.3	9.7	9.7	9.6	9.4	9.2	9.0	9.8	9.7	10.0	9.5 ^b^
1	9.6	8.5	9.1	9.6	9.2	9.6	9.2	8.9	9.5	9.9	10.1	10.2	9.4 ^b^
Mean	9.6 ^a^				9.5 ^bc^				9.4 ^c^				
LSD	0.2												0.2
TA (%citric acid)													
Time-0	0.6												
<0.001	0.56	0.55	0.53	0.52	0.56	0.54	0.51	0.47	0.55	0.52	0.49	0.45	0.52 ^a^
0.01	0.55	0.53	0.51	0.50	0.54	0.51	0.48	0.45	0.54	0.47	0.46	0.43	0.49 ^b^
0.1	0.52	0.50	0.47	0.45	0.51	0.49	0.44	0.41	0.53	0.45	0.43	0.40	0.47 ^c^
1	0.53	0.51	0.49	0.47	0.49	0.47	0.42	0.38	0.50	0.44	0.42	0.37	0.46 ^c^
Mean	0.51 ^a^				0.48 ^b^				0.47 ^b^				
LSD	0.02												0.02
Vitamin C (mg 100 mL^−1^)													
Time-0	7.1												
<0.001	7.1	6.7	6.5	6.3	8.9	10.4	6.7	8.0	10.2	9.1	8.0	7.7	8.0 ^a^
0.01	6.6	6.3	6.0	5.7	7.3	9.7	6.4	6.9	8.6	7.8	7.4	7.2	7.2 ^b^
0.1	6.4	5.8	5.4	5.3	7.0	9.6	6.1	6.6	8.1	7.4	7.1	6.9	6.8 ^c^
1	5.8	5.6	5.4	4.9	6.9	6.2	5.4	5.8	7.0	6.6	5.5	6.3	6.0 ^d^
Mean	6.0 ^a^				7.4 ^b^				7.6 ^b^				
LSD	0.4												0.4

Values are the means of 4 replicates. Means with the same superscript letter across each column and row are not significantly different at *p* ≤ 0.05. TSS: total soluble solids; TA: titratable acidity.

**Table 7:** FRAP activity of ‘Afourer’ mandarins during storage at 20, 10 and 5 °C.

Storage Temperature /Storage Time	FRAP (µM TE 100 mL^−1^)				Mean
(Weeks)	0.001	0.01	0.1	1 µL L^−1^	
20 °C					
Time-0	14.7				
1	10.3	9.8	9.2	8.8	
2	8.6	8.2	8.0	7.3	
3	8.4	7.8	7.5	7.1	
4 weeks	8.2	7.5	7.3	6.8	8.2 ^a^
10 °C					
2	12.5	12.1	11.8	11.7	
4	12.2	11.7	11.5	11.1	
6	11.5	9.5	9.3	9.2	
8 weeks	10.9	8.7	8.3	7.4	10.6 ^b^
5 °C					
2	13.4	13.8	14.2	13.0	
4	12.6	13.0	13.1	11.9	
6	11.7	12.3	12.4	11.1	
8 weeks	10.2	11.3	11.9	9.5	12.2 ^c^
Mean	10.9 ^a^	10.5 ^a^	10.4 ^a^	9.6 ^b^	
LSD	0.6				0.6

Values are the means of 4 replicates. Means with the same superscript letter across each column and row are not significantly different at *p* ≤ 0.05.
